# Comparison of Serum Potassium, MagnEsium, and Calcium Levels between Kanamycin and Capreomycin-BASEd Regimen-Treated MultiDrug-Resistant TuBerculosis Patients in Bandung (CEASE MDR-TB): A Retrospective Cohort Study

**DOI:** 10.1155/2019/5065847

**Published:** 2019-03-14

**Authors:** Arto Yuwono Soeroto, Guntur Darmawan, Rudi Supriyadi, Panji Gugah Bhaskara, Prayudi Santoso, Bachti Alisjahbana, Ida Parwati

**Affiliations:** ^1^Respirology and Critical Illness Division, Internal Medicine Department, Hasan Sadikin General Hospital/Faculty of Medicine, Universitas Padjadjaran, Bandung, Indonesia; ^2^Internal Medicine Department, Hasan Sadikin General Hospital/Faculty of Medicine, Universitas Padjadjaran, Bandung, Indonesia; ^3^Nephrology and Hypertension Division, Internal Medicine Department, Hasan Sadikin General Hospital/Faculty of Medicine, Universitas Padjadjaran, Bandung, Indonesia; ^4^Tropical and InfectiousDisease Division, Internal Medicine Department, Hasan Sadikin General Hospital/Faculty of Medicine, Universitas Padjadjaran, Bandung, Indonesia; ^5^Clinical Pathology Department, Hasan Sadikin General Hospital/Faculty of Medicine, Universitas Padjadjaran, Bandung, Indonesia

## Abstract

Treatment of multidrug-resistant tuberculosis (MDR-TB) with second-line injectable drugs may result in an electrolyte imbalance. This retrospective study was performed to compare and evaluate the effect of kanamycin and capreomycin on serum potassium, calcium, and magnesium in the first and second month treatment at a tertiary, top-referral hospital in Bandung, Indonesia. Data from 84 subjects with complete medical records of at least serum potassium during either kanamycin-based or capreomycin-based treatment were retrieved from the institutional database. Among these, 53 subjects had complete serum calcium data and 53 subjects had complete serum magnesium data. After the first month of MDR-TB treatment, there was a significant decrease in mean serum potassium (4.0 ± 0.4 mEq/L to 3.7 ± 0.5 mEq/L, *p* < 0.003) in the kanamycin-based group and (4.1 ± 0.5 mEq/L to 3.2 ± 0.6 mEq/L, *p* < 0.001) in the capreomycin-based group. Serum potassium levels were significantly lower in the capreomycin-based group than in the kanamycin-based group (3.2 ± 0.6 mEq/L vs 3.7 ± 0.5 mEq/L, *p* < 0.001). The incidence of hospitalization and requirement for a change in the treatment regimen due to electrolyte imbalances were higher in the capreomycin-based group. No previous longitudinal study has evaluated serum potassium, magnesium, and calcium from the first month of MDR-TB treatment with either kanamycin-based or capreomycin-based regimens. Our findings emphasize the importance of routine monitoring of serum potassium, magnesium, and calcium during MDR-TB treatment, and that more attention should be paid when treatment is given using the capreomycin-based regimen. Moreover, our study supported the 2018 World Health Organization treatment guideline recommendations for removal of kanamycin and capreomycin from the MDR-TB regimens.

## 1. Introduction

Multidrug-resistant tuberculosis (MDR-TB) remains a worldwide scourge and presents a major challenge to TB eradication campaigns. It is defined as TB resistant to at least both rifampicin and isoniazid [1]. Inappropriate treatment leads to drug-resistant TB cases, with an estimated 480,000 new cases of drug-resistant TB reported in 2015. There has also been an increase in the percentage of MDR-TB cases in Europe, from 18.3% in 2011 to 22.9% in 2015. Southeast Asia accounts for almost half of the world's TB burden, and Indonesia is one of the top 20 high MDR-TB-burden countries [2–6].

The standard treatment regimen for MDR-TB consists of a combination of first-line and second-line TB drugs to which *Mycobacterium tuberculosis* is still sensitive. Patients in the intensive phase treatment of MDR-TB require at least 4 effective TB medicines. One of the most important drug types other than fluoroquinolones are second-line injectable drugs [7]. In Indonesia, there are 2 widely used second-line injectable drugs: kanamycin and capreomycin. Both of these drugs may affect serum potassium, calcium, and magnesium, even from the first month of treatment. This may result in nonadherence to the treatment and consequent treatment failure [8–10].

Our study aimed at comparing and evaluating the effects of kanamycin and capreomycin on serum potassium, calcium, and magnesium levels during the first and second month of MDR-TB treatment.

## 2. Materials and Methods

### 2.1. Study Design and Study Area

This was a retrospective cohort study conducted at Dr. Hasan Sadikin General Hospital, a tertiary, top referral, university-affiliated teaching hospital in Bandung, Indonesia. The study was approved by the Ethics Committee of Dr. Hasan Sadikin Hospital (Approval number: LB.04.01/A05/EC/053/III/2018), and the need to obtain informed consent from patients was waived due to the retrospective nature of the study.

### 2.2. Study Population

We retrospectively retrieved the complete medical records of adult MDR-TB patients in the MDR-TB clinic of the Dr. Hasan Sadikin Hospital between January 2012 and September 2017. The selection criteria included the following: (1) age ≥ 18 years, (2) MDR-TB diagnosis based on standard diagnosis criteria (Xpert and/or conventional culture) and MDR-TB treatment using a kanamycin-based (15–20 mg/kg daily; maximum dose, 1 g) or capreomycin-based (15–20 mg/kg daily; maximum dose, 1 g) regimen [11–14], and (3) having at least serum potassium examination data available for the initial visit, first month, and second month of treatment. The exclusion criteria were as follows: (1) being pregnant, (2) having chronic kidney disease, thyroid disorder, or using drugs that may affect serum electrolytes, such as angiotensin-converting enzyme (ACE) inhibitors, angiotensin receptor blockers (ARBs), diuretics, steroids, nonsteroidal anti-inflammatory drugs, beta 2 agonists, insulin injections, and tenofovir, and (3) having hypokalemia, hypomagnesemia, or hypocalcemia at the initial visit. Subjects were divided into 2 groups: the kanamycin-based and capreomycin-based regimen groups. Subjects received other MDR-TB drugs, such as fluoroquinolone, cycloserine, ethionamide, and pyrazinamide, concomitantly, according to the MDR-TB treatment guidelines.

### 2.3. Sample Size Calculation

Based on a previous study of the effect on kanamycin on serum potassium [9], we calculated the minimum sample size using the G-Power program. A minimum of 32 subjects per group were required, with a power of 0.95.

### 2.4. Outcome

The occurrence of hypokalemia, hypomagnesemia, or hypocalcemia in each group after initiation of MDR-TB treatment was the main outcome of this study. We also evaluated the incidence of subjects that required hospitalization, shifted to other regimens in the first month or second month of treatment within each group. Laboratory examinations were performed at the clinical pathology department of Dr. Hasan Sadikin Hospital, Bandung, at the initial visit and during the end of the first and end of the second month of treatment. Hypokalemia was defined as serum potassium levels < 3.5 mEq/L [15]. Hypomagnesemia was defined as serum magnesium levels < 1.6 mg/dL [16]. Hypocalcemia was defined as serum calcium ion levels < 4.5 mg/dL [17, 18].

### 2.5. Statistical Analyses

Statistical analysis was conducted using SPSS statistical software. Baseline characteristics of subjects in the kanamycin-based and capreomycin-based regimen groups were compared. Continuous variables with a normal distribution were reported in terms of mean and standard deviation. Continuous variables with a skewed distribution were reported in terms of median, minimum, and maximum values. Comparisons of the means of continuous variables between the kanamycin-based and capreomycin-based groups were performed by using unpaired *t* tests. Comparisons of the medians of continuous variables between the kanamycin-based and capreomycin-based groups were performed by using the Mann–Whitney test. Comparisons of the means of continuous variables among the initial, first, and second months of treatment were performed by using analysis of variance (ANOVA). Comparisons of the medians of continuous variables among the initial, first, and second months of treatment were performed by using the Wilcoxon test. Categorical data were analyzed using Fisher's exact test. A *p* value < 0.05 was considered statistically significant.

## 3. Results

There were 84 subjects with complete medical records of serum potassium analyses. Among them, 53 subjects had complete medical records of serum magnesium and 53 subjects had complete medical records of serum calcium analyses. Among the 84 subjects with complete serum potassium data, 44 subjects were treated with the kanamycin-based regimen and 40 subjects were treated with the capreomycin-based regimen. Among the 53 subjects with complete serum magnesium data, 34 subjects were treated with the kanamycin-based regimen and 19 subjects with the capreomycin-based regimen. Among the 53 subjects with complete serum ion calcium data, 33 subjects were treated with the kanamycin-based regimen and 20 subjects with the capreomycin-based regimen. There was no significant difference in baseline characteristics, such as sex, age, comorbid conditions, concomitant drugs, initial serum potassium levels, and initial serum magnesium levels (Table 1).

There was a significant decrease in mean serum potassium levels within each group during the first month and second month of treatment; hypokalemia was significantly lower in the capreomycin-based group than that in kanamycin-based group during the second month of treatment (Tables 2 and 3). Mean serum magnesium levels tended to decrease in both groups, and this decrease was statistically significant within the kanamycin group (Table 4). Mean serum magnesium levels were lower in the capreomycin-based group than that in the kanamycin-based group during the second month of treatment, but this difference was not statistically significant (Table 5). Mean serum calcium was significantly decreased only within the capreomycin-based group, and there was no significant difference between the 2 groups (Tables 6 and 7).

In the capreomycin-based group, 2 subjects were hospitalized due to electrolyte imbalances in the first month and 1 during the second month; moreover, 1 subject underwent a regimen change from the first to the second month of treatment in the capreomycin-based group. No subject was hospitalized due to electrolyte imbalances or required a change of regimen in the kanamycin-based group.

## 4. Discussion

Second-line injectable drugs are bactericidal antibiotics and include aminoglycoside and polypeptide antibiotics. Kanamycin is the aminoglycoside most frequently used in MDR-TB regimens, while capreomycin is a cyclic peptide antibiotic inhibiting ribosomal protein synthesis. Both drugs are excreted through the kidneys and may result in electrolyte imbalance as an adverse effect due to this renal excretion [10, 13, 19, 20].

Electrolyte imbalances have been reported since the early use of capreomycin [21]. Similar to previous studies by Shin et al., Amalia, and Rahmawati et al., our study demonstrated lower serum potassium levels in the capreomycin-based group than in kanamycin-based group [10, 22, 23]. In contrast to previous studies [9, 10, 22–24], we excluded subjects with any comorbidities and only included subjects with normal baseline serum potassium and magnesium levels, in order to focus on the effects of kanamycin or capreomycin on serum electrolytes. Both groups showed statistically significant decreases in mean serum potassium levels even in the first month of treatment. Moreover, hypokalemia occurred in the capreomycin-based group during the first month of treatment, which was earlier than that reported by Shin et al. [10]. The incidence of severe hypokalemia was higher in the capreomycin-based group than in the kanamycin-based group. Two case reports by Holmes et al. and Sharma and Sahay demonstrated hypomagnesemia in the MDR-TB patients who were treated with a capreomycin-based regimen [21, 25]. A study by Arnold et al. found hypomagnesemia in some subjects within a capreomycin-based treatment group but found no evidence of hypomagnesemia in a kanamycin-based treatment group [24]. Our study revealed that serum magnesium levels tend to decrease in both groups, but it was only statistically significant in the kanamycin-based group; this finding may be due to the small number of subjects in the capreomycin group. We also demonstrated lower serum magnesium levels in the 2^nd^ month of treatment in the capreomycin-based group; additionally, 2 subjects in this group had severe hypomagnesemia. To date, only case studies have reported hypocalcemia in patients treated with the capreomycin-based regimen and the kanamycin-based regimen [21, 25, 26]. In accordance with these previous case reports, our study revealed a decrease in ionized serum calcium in both groups, and this decrease was statistically significant within the capreomycin group. Hypomagnesemia may have contributed to hypocalcemia in both groups.

Kanamycin and capreomycin can cause electrolyte imbalance through stimulation of the calcium-sensing receptor (CaSR) in the thick ascending limb of the loop of Henle. Both drugs, mimicking the effect of extracellular calcium stimulation, inhibit the Na^+^, K^+^, and Cl^−^ symporter channel (NKCC2), renal outer medullary potassium channel (ROMK), Na^+^ and K^+^-ATPase, and/or paracellular diffusion. Moreover, aminoglycosides and capreomycin could induce secondary hyperaldosteronism. These all increase urinary excretion of potassium, magnesium, and calcium, resulting in hypokalemia, hypomagnesemia, and hypocalcemia [9, 10, 16, 27, 28]. As shown in the present study and previous studies, both kanamycin- and capreomycin-based groups had decreased serum electrolytes during treatment and the levels of serum potassium, magnesium, and calcium were lower in the capreomycin-based group than in the kanamycin-based group because capreomycin has a greater number of amino groups than kanamycin, resulting in greater stimulation of the CaSR. Although there was no statistically significant difference in the mean age between the two groups, subjects in the capreomycin-based group were older than those in the kanamycin-based group. This may be due to the higher frequency of hearing problems in older people, which favored the use of the capreomycin-based regimen. As shown in the study by Sagwa et al., kanamycin use had higher hearing impairment than that of capreomycin use [29]. Age might also play a role in electrolyte imbalance, due to decreased renal function [10, 15]. In previous studies, the impact of hypokalemia was varied, with death as the most severe consequence. Altered electrolyte levels may result in deadly arrhythmias. Some agents used in MDR-TB regimens such as quinolones can prolong the QT interval, and using either kanamycin or capreomycin might be even more dangerous in this clinical condition [10, 22]. In our study, there was no mortality, but some subjects in the capreomycin-based group were hospitalized due to electrolyte imbalances, and their regimens were consequently changed.

Moreover, in 2018, the World Health Organization (WHO) no longer included either kanamycin or capreomycin in MDR-TB treatment regimens, due to the increased risk of poor outcomes [30]. The result in our study supported this recommendation, and national guidelines need to be revised.

No previous longitudinal study evaluating serum potassium, magnesium, and calcium during MDR-TB treatment in both kanamycin-based and capreomycin-based regimens has been reported to date. Moreover, our study included only subjects with no other comorbid diseases or who were taking medications that may affect serum potassium, magnesium, and calcium levels at baseline. Subjects with electrolyte disorder usually require supplementation, which consequently influence the serum electrolyte levels. Therefore, our study excluded subjects with hypokalemia, hypomagnesemia, or hypocalcemia at baseline.

Our study had several limitations. First, there was no adjustment for dietary factors that might influence serum potassium, magnesium, and calcium levels. Second, some TB drugs might induce nausea, vomiting, or diarrhea, which can alter electrolyte levels. We did not have access to records of these adverse effects in our study. Third, since serum calcium and magnesium were not routinely examined, many subjects did not have complete data on serum calcium and magnesium levels during treatment. This might have resulted in a bias due to a reduced data set. Fourth, we did not measure the effect of vitamin D on serum calcium levels. Fifth, we did not examine plasma aldosterone levels and thus could not rule out secondary hypoaldosteronism as a cause of an electrolyte imbalance. We also did not have any data on blood gas, or serum sodium levels, which may provide more information about the magnitude of the effect of kanamycin-based and capreomycin-based regimens. Further studies involving a greater number of subjects, with a prospective design, and evaluating more electrolytes, such as sodium, are thus warranted.

## 5. Conclusion

Our study underlined the importance of routine monitoring of serum potassium, magnesium, and calcium levels, starting from the first month of MDR-TB treatment involving either kanamycin-based or capreomycin-based regimens and showed a greater electrolyte imbalance in the capreomycin-based group. Our study findings supported the 2018 WHO recommendation for removal of kanamycin and capreomycin from MDR-TB treatment regimens.

## Figures and Tables

**Table 1:** Baseline characteristics among 84 patients receiving MDR-TB treatment.

	Kanamycin (*n*=44)	Capreomycin (*n*=40)	*p* value
Sex			
Male, *n* (%)	33 (75.0)	29 (72.5)	0.795^a^
Age (years)			
Mean ± SD	39 ± 11	41 ± 13	0.346^b^
Comorbid, *n* (%)			
Yes	5 (11.4)	2 (5.0)	0.437^c^
No	39 (88.6)	38 (95.0)	
Concomitant drugs, *n* (%)			
Yes	3 (6.8)	2 (5.0)	1.000^b^
No	41 (93.2)	38 (95.0)	
Serum potassium	4.0 ± 0.4 mEq/L	4.1 ± 0.5 mEq/L	0.100^b^
Serum magnesium	2.33 ± 0.21 mg/dL	2.23 ± 0.18 mg/dL	0.082^b^
Serum calcium	4.80 ± 1.04 mg/dL	4.74 ± 0.37 mg/dL	0.839^b^

Data are given as mean ± SD or number (%). *p* values are determined using ^a^chi-square test, ^b^*t*-test, and ^c^Fisher's exact test.

**Table 2:** Comparison of serum potassium values at the initial visit, first month, and second month of treatment within the kanamycin-based and capreomycin-based groups.

		Initial visit Mean ± SD (mEq/L)	First month Mean ± SD (mEq/L)	Second month Mean ± SD (mEq/L)	*p* value
Kanamycin	*N*	44	44	44	**<0.001** ^a^
	Potassium	4.0 ± 0.4	3.6 ± 0.4	3.7 ± 0.5	
	Difference	—	−0.3	−0.3	
	*p* value	—	**<0.001** ^b^	**0.003** ^b^	

Capreomycin	*N*	40	40	40	**<0.001** ^a^
	Potassium	4.1 ± 0.5	3.4 ± 0.7	3.2 ± 0.6	
	Difference	—	−0.7	−0.9	
	*p* value	—	**<0.001** ^b^	**<0.001** ^b^	

SD, standard deviation; analyzed using ^a^one-way ANOVA with post hoc ^b^Bonferroni test.

**Table 3:** Comparison of serum potassium values between the kanamycin-based group and the capreomycin-based group at the initial visit, first month, and second month of treatment.

		*N*	Potassium		*p* value
			Mean ± SD (mEq/L)	Min-max (mEq/L)	
Initial visit	Kanamycin	44	4.0 ± 0.4	3.3–4.8	0.100
	Capreomycin	40	4.1 ± 0.5	3.5–5.5	

First month	Kanamycin	44	3.6 ± 0.4	2.8–4.3	0.086
	Capreomycin	40	3.4 ± 0.7	1.8–4.7	

Second month	Kanamycin	44	3.7 ± 0.5	2.4–4.7	**<0.001**
	Capreomycin	40	3.2 ± 0.6	1.8–4.2	

SD, standard deviation; min, minimum value; max, maximum value; analyzed using the unpaired *t*-test.

**Table 4:** Comparison of serum magnesium values at the initial visit, first month, and second month of treatment within the kanamycin-based and capreomycin-based groups.

		Initial visit Mean ± SD (mg/dL)	First month Mean ± SD (mg/dL)	Second month Mean ± SD (mg/dL)	*p* value
Kanamycin	*N*	34	34	34	**<0.001** ^a^
	Magnesium	2.33 ± 0.21	2.11 ± 0.20	2.11 ± 0.23	
	Difference	—	−0.22	−0.22	
	*p* value	—	**<0.001** ^b^^*∗*^	**<0.001** ^b^^*∗*^	

Capreomycin	*N*	19	19	19	0.083^a^
	Magnesium	2.23 ± 0.18	2.13 ± 0.34	1.96 ± 0.51	
	Difference	—	−0.10	−0.27	
	*p* value	—	0.814^b^	0.056b	

SD, standard deviation; analyzed using ^a^one-way ANOVA with the post hoc ^b^Bonferroni test.

**Table 5:** Comparison of serum magnesium values between the kanamycin-based group and the capreomycin-based group at the initial visit, first month, and second month of treatment.

		*N*	Magnesium		*p* value
			Mean ± SD (mg/dL)	Min-max (mg/dL)	
Initial visit	Kanamycin	34	2.33 ± 0.21	1.80–2.75	0.082
	Capreomycin	19	2.23 ± 0.18	1.94–2.58	

First month	Kanamycin	34	2.11 ± 0.20	1.63–2.55	0.883
	Capreomycin	19	2.13 ± 0.34	1.30–2.55	

Second month	Kanamycin	34	2.11 ± 0.23	1.51–2.60	0.183
	Capreomycin	19	1.96 ± 0.51	0.80–2.73	

SD, standard deviation; min, minimum value; max, maximum value; analyzed using the unpaired *t*-test.

**Table 6:** Comparison of serum calcium values at the initial visit, first month, and second month of treatment within the kanamycin-based and capreomycin-based groups.

		Initial visit Mean ± SD (mg/dL)	First month Mean ± SD (mg/dL)	Second month Mean ± SD (mg/dL)	*p* value
Kanamycin	*n*	33	33	33	0.367^a^
	Calcium	4.80 ± 1.04	4.56 ± 0.39	4.65 ± 0.45	
	Difference	—	−0.24	−0.15	
	*p* value	—	0.324	0.762	

Capreomycin	*n*	20	20	20	**0.009** ^a^
	Calcium	4.74 ± 0.37	4.50 ± 0.23	4.43 ± 0.35	
	Difference	—	−0.24	−0.31	
	*p* value	—	**0.044** ^b^	**0.007** ^b^	

SD, standard deviation; analyzed using the ^a^one-way ANOVA test with the post hoc ^b^Bonferroni test.

**Table 7:** Comparison of serum calcium values between the kanamycin-based group and the capreomycin-based group at the initial visit, first month, and second month of treatment.

		*N*	Calcium		*p* value
			Mean ± SD (mg/dL)	Min-max (mg/dL)	
Initial visit	Kanamycin	33	4.80 ± 1.04	3.89–10.33	0.839
	Capreomycin	20	4.74 ± 0.37	4.26–5.61	

First month	Kanamycin	33	4.56 ± 0.39	3.69–5.16	0.434
	Capreomycin	20	4.50 ± 0.23	4.05–4.94	

Second month	Kanamycin	33	4.65 ± 0.45	3.61–5.33	0.053
	Capreomycin	20	4.43 ± 0.35	3.65–4.98	

SD, standard deviation; min, minimum value; max, maximum value; analyzed using the unpaired *t*-test.

## Data Availability

All data arising from this study are present within the manuscript.
